# Supplementary material for: Molecular mechanism of exchange coupling in CLC chloride/proton antiporters
Source: Nat Commun. 2026 Jan 8;17:1342. doi: 10.1038/s41467-025-68098-1 (PMC12873427; doi:10.1038/s41467-025-68098-1)
Supplement: Supplementary file 2 — Description of Additional Supplementary Files [file 41467_2025_68098_MOESM2_ESM.pdf]

Description of Additional Supplementary Files

File Name: Supplementary Movie 1

Description: Deprotonated E_{gate} expels Cl^- to the outside. A close-up view of the Cl^- pathway, as seen from the membrane perspective. Inner-gate residues S017 and Y445, along with outer-gate residue E_{gate}, are depicted as stick models. Cl^- ions are represented as teal spheres. As E_{gate} moves into the Cl^- pathway, concomitant opening of the inner gate positions the transporter to allow Cl^- entry from the intracellular side.
